# Features of Human Decidual NK Cells in Healthy Pregnancy and During Viral Infection

**DOI:** 10.3389/fimmu.2019.01397

**Published:** 2019-06-28

**Authors:** Nabila Jabrane-Ferrat

**Affiliations:** ^1^CNRS UMR 5282, Center of Pathophysiology Toulouse Purpan, Toulouse, France; ^2^INSERM UMR1043, Purpan University Hospital, Toulouse, France; ^3^Toulouse III University, Toulouse, France

**Keywords:** pregnancy, decidual natural killer, congenital infection, receptor, cytokines

## Abstract

The hallmark of human early pregnancy is the accumulation of a unique population of Natural Killer (dNK) cells at the main maternal-fetal interface, the *decidua basalis*. dNK cells play a crucial role in successful placentation probably by orchestrating the invasion of trophoblast cells deep into the *decidua basalis* and remodeling of the maternal spiral arteries. Recent advances in the field emphasize the importance of the local microenvironment in shaping both the phenotype and the effector functions of these innate lymphoid cells. Despite slow progress in the field, *ex vivo* studies revealed that dNK cells sense and destroy infected cells in order to protect the fetus from invading pathogens. In this review, we will discuss key features of dNK cells during healthy pregnancy as well as their functional adaptations in limiting pathogen dissemination to the growing conceptus. The challenge is to better understand the plasticity of dNK cells in the maternal-fetal interface. Such insights would enable greater understanding of the pathogenesis in congenital infections and pregnancy disorders.

## Immunological Paradox of Human Pregnancy

Seven decades ago, Sir Peter Medawar wondered: “How does the pregnant mother contrive to nourish within itself, for many weeks or months, a fetus that is an antigenically foreign body?” (1). This interrogation highlighted the immunological paradox of pregnancy. Ever since, the compelling relationship between two mismatched individuals, the mother and her fetus, prompted the development of a novel reproductive immunology research stream. The original theories claiming the antigenic immaturity of the fetus, inertness of the maternal immune system, and the presence of an anatomical barrier between the embryo and its mother have proven wrong. As a result, the modern concept of active immune crosstalk emerged. Recent advances in the field advocate a unique bidirectional immune dialogue involving the fetus and mothers' innate as well as adaptive immune cells; namely innate lymphoid cells, regulatory T cells, macrophages, and dendritic cells (2–7). In this review, we discuss the current understanding of how a unique population of type 1 innate lymphoid cells (ILC-1), the uterine Natural Killer cells found at the maternal *decidua basalis* (called hereafter dNK cells), supports the development of the fetal placenta while maintaining active immune surveillance against invading pathogens.

## Human Pregnancy

Every month, the uterine mucosa or endometrium undergoes singular anatomical changes, the most crucial ones occurring during pregnancy. Implantation of the semi-allogeneic blastocyst is synchronized with massive adaptations of the uterine mucosa which transforms into the *decidua basalis*. The blastocyst produces large amounts of the chorionic gonadotrophin hormone (CGH) to maintain high levels of progesterone. These hormonal changes prevent menstruation, destruction of the decidualized endometrium, regulate immune cell functions, and promote angiogenesis. Other factors, such as the leukemia inhibitory factor (LIF), IL-6, and matrix metalloproteinases, are also highly expressed during the implantation process.

In humans, the embryo is completely embedded within the endometrium and the implantation is termed interstitial hemochorial. The development of the placenta is initiated with the apposition of the trophectoderm layer of the blastocyst to the uterine mucosa. The rapid proliferation of this extra-embryonic cell layer generates a unique type of placental cell, the trophoblast, which will further develop into the floating and anchoring chorionic villi of the placenta. The underlying stromal core of the placental villi originates from the extraembryonic mesoderm. The proliferative cytotrophoblasts (CTBs) follow two differentiation programs (8–12). In the first program, CTBs fuse to form the syncytiotrophoblast (STB), a multinucleated epithelial outer layer of the floating chorionic villi. The STBs, in direct contact with maternal blood, ensure nutrient, and gas exchanges for the conceptus. In the second program, CTBs in the cell column of the anchoring villi differentiate into extravillous trophoblasts (EVTs). CTBs and EVTs exhibit differential expressions of cell adhesion molecules, integrins, growth factors as well as the immune inhibitory molecules Fas Ligand, TRAIL, and Indoleamine 2,3-dioxygenase (IDO) (13). Consistent with the role of some of these factors in dampening T cell response, it is possible that EVTs contribute to fetal tolerance.

**Figure 1:**
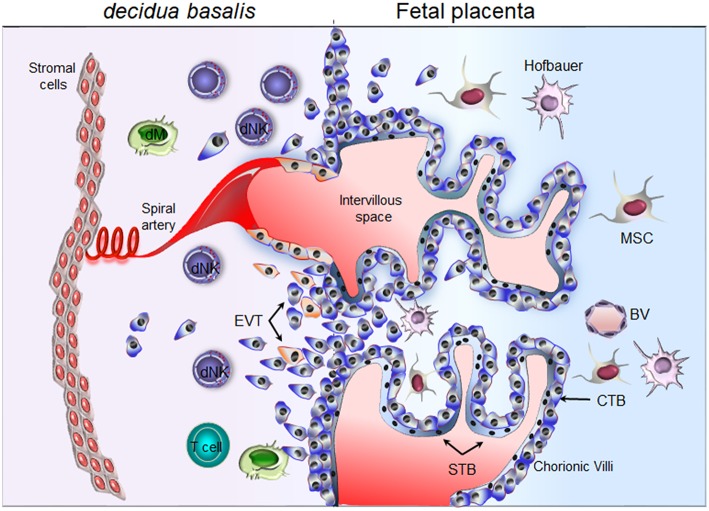
Schematic representation of the maternal-fetal interface. Floating chorionic villi are bathed in maternal blood within the intervillous space. A multinucleated syncytiotrophoblast (STB) outer cell layer covering the chorionic villi. STB layer serves for transport of nutrient and barrier function. A layer of cytotrophoblast cells (CTBs), underlines the STB. CTBs differentiate into extravillous trophoblast (EVTs) and invade the maternal *decidua*. Through the release of soluble factors (cytokines, chemokines, and proangiogenic factors), maternal decidual NK (dNK) cells participate actively in the attraction of invasive EVTs and remodeling of the spiral arteries. Invasive EVTs are also in contact with decidual macrophages (dM) and T cells. Fetal blood vessel (BV), mesenchymal stem cells (MSC), Hofbauer cells (fetal macrophage).

Unlike most cells of the body, EVTs express only the less polymorphic HLA-C and non-classical HLA-E and HLA-G molecules (14, 15). It is believed that these HLA molecules mediate recognition of invading EVTs by maternal dNK cells rather than T cells (16, 17). The recognition of HLA-G molecule and HLA-G peptides presented in the context of HLA-E may contribute to NK cell hyporesponsive (18–20). Even if the detailed mechanisms of EVT invasion of the placental bed are still largely unknown, progressive remodeling of maternal spiral arteries by EVTs seems to follow two separate waves (21, 22). In the first 10–12 weeks, endovascular migration and plugging of the maternal arteries prevents blood flow to the intervillous space and creates a hypoxic environment that is necessary for placental and fetal development (23, 24). These original claims were confirmed by *in vivo* monitoring of the oxygen tension at different gestational ages (25, 26). The second wave of EVT invasion, starting around 14 weeks, stops at the inner myometrium. The resulting intramural incorporation of invasive EVTs into the vessel wall and erosion of the trophoblastic plug are needed to establish proper blood flow to the intervillous space of the developing placenta (27–29). These early and late developmental steps result in the establishment of privileged sites, where embryonic trophoblasts intermingle with maternal cells. The best examples are the decidua, hosting a large number of innate immune cells in early pregnancy, and the intervillous space, where maternal blood bathes the chorionic floating villi (Figure 1). Flaws in EVT invasion and arteries' remodeling can lead to placental dysfunction and major pregnancy disorders such as preeclampsia, fetal growth restriction (FGR) and recurrent miscarriage (30).

## Natural Killer Cells

Natural killer (NK) cells are cytotoxic innate lymphoid cells known for their active role in immune regulation of leukocyte activation and immune surveillance of microbial infections and malignancies (31–33). Human conventional NK cells in peripheral blood (cNK/pNK/) have been extensively studied in health and disease. NK cells were regarded as innate immune cells, owing to the lack of expression of antigen-specific receptors. NK cell responsiveness is governed by the diversity of their germline encoded activating and inhibitory receptors (NKR). Originally, cNK cells were subdivided into two main subsets; the CD56^dim^CD16^+^ cytotoxic cells and the CD56^bright^CD16^−^ cytokine producer cells. However, recent developments in the field regarding NK cell educational programs and the diversification of NKR in response to pathogens, as well as the development of memory-like capacities, suggest the existence of more than two NK cell subsets (34).

Similar to the periphery, distinct subsets of resident NK cells (trNK) have been found in many tissues including the liver, kidney and uterus (35). While trNK share striking similarities with cNK cells, these CD56^bright^ cells exhibit different signatures that are related to their tissue of origin. Similar to tissue-resident T cells, trNK express high levels of CD69, CD103, and CD49a (2, 3, 36–38). Here, we will mainly focus on the aforementioned dNK cells that reside in the decidua.

## Pregnancy and Immunity: Regulators of the Maternal-fetal Interface

Decidualization requires coordinated contribution of the uterine glands, stromal cells, and immune cells (4, 39–41). In early pregnancy, the hallmarks of the decidua include the accumulation of immune cells that represent up to 40% of total decidual cells and the histiotrophic nurturing of the developing placenta by the uterine glands. The distinctive Tbet^pos^EOMES^pos^CD56^bright^ dNK cell population accounts for almost 70% of total tissue leukocytes (42, 43). Whereas, T cells account for ~5–10% of total leukocytes, the quasi absence of B or plasma cells suggests it is very unlikely that any antibody response would harm the invading EVTs (44–46). Additional innate immune cells include CD14^pos^ macrophages and dendritic cells, which represent ~20%. Besides dNK cells, other ILCs are found at the implantation bed including a non-NK ILC1 subset as well as both NCR^+^ and NCR^−^ ILC3 (43, 47, 48). These decidual ILCs share similarities with other tissue resident ILCs. Upon *in vitro* stimulation, decidual ILC1 are able to produce IFN-γ while NCR^+^ILC3 produce IL-22 and IL-8 and NCR^−^ILC3 produce TNF and IL-17 (43, 47, 48). Finally, in addition to the typical T cell populations (CD8, CD4, γδT cells), the non-pregnant uterine mucosa and first trimester decidua contain a small fraction of mucosal-associated invariant T (MAIT) cells [(49) and unpublished data from our laboratory]. Yet, the exact functional role of decidual ILCs and MAIT cells in pregnancy is not clear.

### Decidual NK Cells

The discovery of dNK cells at the implantation site, even before the implantation of the blastocyst, has led to the idea that these cells play a crucial role in normal placentation (50). As a matter of fact, the uterus is undeniably among the peripheral organs that exhibit the highest frequency of NK cells. After ovulation, the surge of IL-15 and prolactin, triggered by the exposure of stromal cells to progesterone, induces a rapid proliferation and differentiation program of uterine NK cells (51). These numbers increase further when implantation is successful and are maintained throughout the second trimester. dNK cell numbers decline from mid-gestation onward to reach a barely detectable level at term. Despite extensive work on dNK cells, we are still lacking essential information about their origin and exact functions. The association of dNK cells with EVTs and their spatiotemporal localization at the vicinity of maternal arteries suggest that these immune cells provide a well-balanced microenvironment to enable proper development and functioning of the placenta yet preclude excessive trophoblast invasion.

Research, performed by several groups has yielded fascinating insights into the phenotype and functional plasticity of dNK cells. In contrast to cNK, dNK cells are poorly cytotoxic and display a unique repertoire of NKR (2–4, 9, 38, 52–54). dNK cells are mainly CD56^bright^CD16^−^KIR^+^ cells but they are distinct from the CD56^bright^ subset found in peripheral blood, both at the functional and phenotypical levels. dNK cells express the tissue residency markers CD69, CD49a, integrin β7, and CD9. Additionally, dNK cells express most of the NKRs including NKp46, NKp80, NKG2D, CD94/NKG2A. Contrary to cNK, the CD94/NKG2C heterodimer and NKp44 receptor are found on a fraction of dNK cells (2–4, 38, 52), although other reports demonstrated no expression of NKp44 only freshly isolated cells (55). Nonetheless, similar to cNK, NKp44 expression can be induced on the large population of dNK cells upon *in vitro* stimulation. 2B4 and LILRB, which is expressed at low frequency, act as inhibitory receptors (54, 55). Likewise, freshly isolated unstimulated dNK cells express inhibitory isoforms of the NKp44 and NKp30, natural cytotoxicity receptors 2 and 3 respectively (3). Furthermore, several chemokine receptors including CXCR3, CXCR4, CCR1, and CCR9 are expressed by these cells (3, 53, 56). Fine analysis of the killer-cell immunoglobulin-like receptors (KIR) has highlighted a skewed repertoire toward the recognition of the less polymorphic HLA-C, the only classical HLA class I molecule expressed on EVTs (14). However, several of these NKR are expressed only by a fraction of cells, suggesting that dNK cells may come in different flavors. As an example, the majority of dNK cells express the CD49a residency marker and the chemokine receptor CXCR3, whereas a sizeable fraction of cells lacks the expression of CD103 or CXCR4 (3).

The recent discovery of three main pools of dNK cells (dNK1, dNK2, and dNK3) with different immunomodulatory profiles confirmed these findings (57). The dNK1 pool expresses regulatory CD39 ecto-ATPase, which is involved in shifting the balance from a pro-inflammatory to an immunosuppressive environment [reviewed in (58)] and has high levels of the inhibitory as well as activating KIRs (KIR2DL1, KIR2DL2, KIR2DL3, KIR2DS1, and KIR2DS4). Furthermore, the expression of the high affinity receptor for the HLA-G dimer, *LILRB1*, and the active glycolytic metabolism allude to the interaction of the dNK1 subset with fetal EVTs. The dNK2 pool is characterized by the expression *ANXA1* and *ITGB2*. Both dNK1 and dNK2 cell subsets express the activating *NKG2C* and *NKG2E* and inhibitory *NKG2A* receptors. The third subset expresses *CD160, CD161, TIGIT, CD103*, and *ITGB2*. This unbiased reconstruction of the fetomaternal interface further highlights key interactions between dNK cell subsets, invading fetal EVTs and decidual stromal cells (DSC), all needed for the development of embryonic tissues and successful pregnancy. Whether dNK cell subsets with defined characteristics exert common or distinctive functions within the decidual microenvironment is yet to be defined. dNK cell subsets shall either promote or restrain EVT invasion. However, doubt subsists as to whether this would occur through specific ligand–receptor interaction, metabolic adaptations and expression of checkpoint inhibitors or through microenvironment paracrine effect. While additional studies are required to define the exact function of the three dNK cell subsets, it is clear that maternal adaptations, during pregnancy, are designed to restrain harmful dNK cell responses. Thus, a finely tuned dialogue between a given dNK cell pool, decidual cells and invading EVTs, is necessary for the establishment of the fetomaternal interface and for the development of the placenta and fetus.

### Origin of dNK Cells

**Figure 2:**
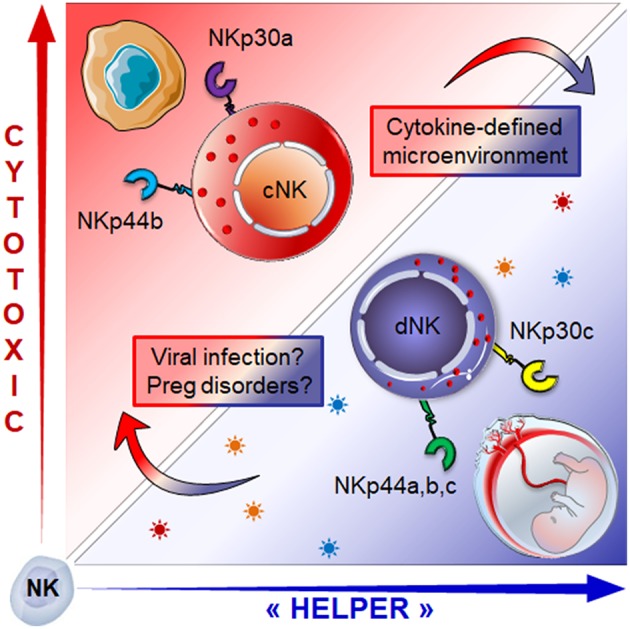
Role of the microenvironment in shaping NK cell phenotype and functions: cNK cells expressing activating isoforms of the NKp30 and NKp44 receptors (NKp30a, NKp44b) which endows them with cytotoxic function, can be converted into dNK like cells. Exposure to a combination of TGF-β, IL-15 and IL-18) drives the isoform expression profile toward regulatory profile (NKp30c, NKp44a,c), a hallmark of dNK cells. This conversion is associated with the switch from cytotoxic to “helper-like” or tolerogenic effector functions associated with major changes in the secretome. How micro-environmental changes during pregnancy disorders or congenital infection might influence the dNK cell plasticity and effector functions is still an open question.

The origin of dNK cells remains subject to controversy. The discovery of CD34^pos^ progenitors in the maternal *decidua* would suggest that dNK cells originate from local self-renewed CD34^+^ progenitors. This perspective is supported by the ability of CD34^pos^ progenitors from the decidua to differentiate into dNK-like precursors, in the presence of DSC and an IL-15-enriched microenvironment (59, 60). The second possible explanation would be that dNK cells arise from NK precursors, as advocated by the presence of CD34^neg^CD117^pos^CD94^neg^ NK cell precursors within the uterine mucosa (61). Lastly, dNK cells could originate from cNK cells recruited from the periphery through chemotaxis (59, 60, 62–64). This latter insight is supported by a twofold argumentation; (i) both estrogens and progesterone induce the secretion of CXCL10, CXCL12, CCL2, CXCL8, and CX3CL1 chemokines by DSC and endothelial cells, ensuring the availability of several chemoattractant axes that can promote the recruitment of cNK cells, and (ii) the conversion of cNK cells in the presence of the transforming growth factor-β (TGF-β) or a combination of TGF-β/IL-15 or yet again, TGF-β/5-aza-2′-deoxycytidine into less cytotoxic cells that can promote the invasion of human trophoblast (65–67). In line, we have shown that cytokines enriched within the decidual microenvironment (68–70), namely TGF-β and IL-15, in combination with IL-18 convert the NKp30/NCR3 and NKp44/NCR2 splice variant profile of cNK cells into one similar to that of dNK cells (3). The switch from activating to inhibitory isoforms of NKp44 and NKp30 was associated with decreased cytotoxic function and major adaptations of NK cell secretome, the two hallmarks of the decidual phenotype (Figure 2).

Thus, whether recruited or tissue resident cells, dNK cells are undoubtedly different from other CD56^bright^ NK cell subsets found in the periphery (2). Today, it is undeniably admitted that microenvironment pressure within the *decidua basalis* conditions the education and the generation of dNK cells with unique phenotypic and functional features; a great ability to produce large amounts of soluble factors and a finely tuned cytotoxic function that are both necessary for a successful pregnancy.

## dNK Cells in Healthy Pregnancy

### dNK Cell Effector Functions

Large scale profiling of dNK cell transcriptome and secretome revealed that these cells produce: (i) a large array of cytokines including IFN-γ, TNF-α, GM-CSF, TGF-β, and IL-10; (ii) chemokines including CXCL8 (IL-8), CCL3 (MIP1a), CCL4 (MIP1b), CCL5 (Rantes), CXCL10 (IP-10), and CXCL12 (SDF-1); and (iii) angiogenic factors including Ang-2, PLGF, EGF, VEGF-A, but also VEGF-C that can induce the expression of inhibitory ligands on trophoblasts (4, 56). Yet, most of these *in vitro* studies were conducted under IL-2 or IL-15 stimulation. In our hands and in agreement with single cell transcriptomic analysis, freshly isolated and unstimulated dNK cells barely produce any IFN-γ or VEGF-A (38, 57). With regard to the cytotoxic effector function, dNK cells express functional activating receptors and a payload lytic machinery including granzymes, granulysin, and perforin, but conversely they lack cytolytic activity in healthy pregnancies (2, 3, 52, 71, 72). Defaulting assembly of the immunological synapse and failure of 2B4 receptor to convey activating signals have been proposed as mechanisms that can explain the poor cytotoxic function of dNK cells (54, 71). Nevertheless, these cells are probably educated in the decidua by the binding of their NKG2A and/or KIR to their cognate ligands expressed by the fetal trophoblast cells, they are also highly plastic and can acquire cytotoxic functions upon NKp46 receptor engagement and/or cytokine stimulation [reviewed in (2)].

### dNK Cells Control Trophoblast Invasion

The tight regulation of EVT invasion into the maternal decidua is essential for the development of the placenta and the outcome of the pregnancy. Inadequate invasion can have disastrous consequences and result in pathological pregnancy such as preeclampsia, FGR, preterm labor, and recurrent miscarriage. The presence of dNK cells in the vicinity of invasive fetal trophoblasts and spiral arteries is suggestive of their active role in regulating the extent of trophoblast invasion and vascular remodeling. The production of a large panel of soluble factors is the likely mechanism for dNK cells to regulate trophoblast invasion. For instance, secreted CXCL8 and CXCL10 bind to their receptors on invasive trophoblasts and promote trophoblast migration while Ang-2, TNF, and TGF-β inhibit trophoblast invasion (56, 73–76). Whether through specific education programs, direct receptor-ligand engagement or paracrine factors, dNK cells contribute grandly to the appropriate trophoblast invasion (72, 77–79). Further elucidation of how the education program shapes dNK cell functions will probably have upshots in resolving some pregnancy disorders.

### dNK Cells Direct Vascular Remodeling

The remodeling of decidual tissue is mandatory to pregnancy success to ensure minimal vessel resistance and high blood flow of nutrients as well as oxygen to the growing conceptus. Even if there are still a lot of controversies regarding different steps of the vascular remodeling process, the invasive EVTs are very like to have an active role in the removal of the smooth muscle media and in the replacement of the endothelium lining deep into the endometrium by mural trophoblast (28, 80, 81). Although lessons from mouse studies highlight the contribution of dNK cells to this process (82), their role is not yet fully elucidated in humans. However, their accumulation along the vascular wall of the changing vessels before endovascular invasion and their production of angiogenic factors is suggestive of an active role in angiogenesis (52, 56). Similar to the extent of trophoblast invasion, specific KIRs express on dNK cells may dictate the fate of vascular remodeling and thus conduct to successful or pathological pregnancies (79, 83).

### Do Decidual NK Cells Remember Pregnancy?

While NK cells were considered as short lived cells for many years, accumulating evidence indicate that cNK cells develop long-lasting memory-like phenotype to viruses marked by high cytotoxicity and characterized by the expression CD94/NKG2C and the CD57 terminal differentiation marker (34, 84). Whether dNK cells develop memory-like phenotypes to pregnancy is still a major debate. Nevertheless, efficient development of the fetal placenta in subsequent pregnancies hints to the existence of a “trained” uterine immunity (85). Pioneer work showing the association between maternal activating KIRs expressed on dNK cells and protection against reproductive failure mediated by fetal HLA-C2 (83), suggest that fine-tuning of dNK responsiveness is necessary for successful pregnancy. Later studies provided evidence that dNK cell response is orchestrated by functional education and expression of inhibitory receptors (86). Whether education takes place even before embryo' implantation and how lessons from first pregnancy shape the uterine immune landscape remain to be elucidated.

Recently, the group of Ofer Mandelboim reported the existence of a specific population of NKG2C^high^LIRB1^+^ dNK cells in the decidua of multigravid women (87). The co-expression of NKG2C and the high affinity receptor for HLA-G dimer suggest that these “trained” dNK cells belong to dNK1 subset (57). The ability of IL15-primed “memory-like” dNK cells to produce high amounts of IFN-γ and VEGF-A upon ligation of NKG2C/E and LIRB1 receptors (87) is a major difference with other “memory” NK cells that produce only IFN-γ. Previous work has clearly established that the physiological pool of dNK cells is governed by the differential expression of NKp30 and NKp44 alternatively spliced isoforms (3). The expression of inhibitory isoforms would act as a secondary innate immune checkpoint that conveys dNK cells with a “support” function contributing to proper placental development and successful pregnancy, while activating isoforms trigger NK cell responsiveness and effector functions. Whether this has physiological relevance to dNK cell training and “memory-like” development is yet to be depicted and warrants further investigations. The development of “memory-like” dNK cells in subsequent pregnancies may explain why deficient placentation are less frequent in subsequent pregnancies. dNK cell hyporesponsiveness in first pregnancies might lead to deficient placentation and pregnancy disorders. Elucidating the potential role of “trained” dNK cell immunity will constitute a new step toward a better understanding of the pathophysiology of pregnancy disorders and the development of new therapeutic intervention.

## dNK Cells During Viral Infections

While many aspects of the immune response are circumvented at the fetomaternal interface to enable an intimate relationship between maternal and fetal cells, many threatening pathogens can jeopardize the harmony of this immune-friendly site. For instance, the growing family of TORCH pathogens which originally includes *Toxoplasma gondii*, other (syphilis, varicella-zoster, parvovirus B19, and others), rubella virus, cytomegalovirus (HCMV) and herpes simplex virus can cause severe maternal and fetal morbidity during pregnancy. Today, the genotype 1 of Hepatitis E virus (HEV-1) and Zika virus (ZIKV) can also be classified as TORCH pathogens (88–92). However, how these viruses reach the developing placenta is still largely unknown and requires active investigations. Lessons from *ex vivo* studies demonstrate that some of these viruses (HCMV, ZIKV, and HEV-1) can use the fetomaternal interface as a replication platform before spreading to the placenta and fetal compartment.

The human cytomegalovirus (HCMV) is a member of the largest virus specie, Betaherpesviridae, with a DNA genome encoding more than 150 proteins (93). HCMV is the most common cause of congenital infections with severe and permanent birth sequelae (94, 95). Even if the transmission rate is much higher in the third trimester, primary infection in the first trimester is associated with high risk of placental pathology and severe congenital syndrome. *Ex vivo* studies demonstrated that replication of HCMV strains in stromal and placental cells results in impaired function and soluble factor secretion (38, 96–99).

The hepatitis E virus (HEV) is a single-stranded RNA virus with five genotypes that can cause acute self-limiting illness in immunocompetent host. During pregnancy, the outcome of infection is quite devastating in some endemic areas where HEV-1 prevales. In fact, HEV-1 infection is associated with a high co-morbidity rate in pregnant women from northern India, due to fulminant hepatic failure associated with severe placental diseases (100, 101). Retrospective studies have estimated vertical transmission in 23–50% of North India cases. However, the regional differences in the course of congenital HEV-1 infection remain unclear. It is highly possible that both environmental and viral factors may contribute to the devastating pregnancy outcome. To provide insights into the genotype-specific pathogenicity of HEV during pregnancy, we *ex vivo* modeled the pathological HEV-1 and less-pathological HEV-3 infection at the maternal-fetal interface using organ cultures of first trimester decidua and fetal placenta. While both HEV genotypes are able to infect the maternal-fetal interface, HEV-1 replicates more efficiently in the decidual and placental tissues as well as in primary isolated stromal cells. The dysregulation of the cytokine microenvironment by HEV-1 caused severe damage to the decidual and fetal placenta tissues (89).

Zika virus (ZIKV) is a mosquito-borne Flavivirus initially isolated in the Zika forest in Uganda in 1947. The recent epidemic wave of ZIKV in the Americas revealed an unprecedented association with a severe congenital syndrome (102, 103). Investigations using decidua and placenta explants have demonstrated that ZIKV replicates in a wide range of cells. In the basal decidua, ZIKV targets EVTs as well maternal macrophages and stromal cells. In the anchoring villi, ZIKV targets the proliferative CTBs and stromal cells of the villous core (90, 91, 104, 105), but not STBs owing to intrinsic antiviral defense mechanisms involving IFN-λ (106). Thus, ZIKV should either overcome the STB restriction mechanisms or exploit alternative strategies to access the fetal compartment.

To date, our understanding of how viruses reach the fetal compartment and whether they exploit common infection routes is still in its infancy. Usually, the vertical transmission rate is quite low in the first trimester of pregnancy, which coincides with high numbers of dNK cells within the placental bed. Whether these immune cells are able to restrain viral spread at the maternal-fetal interface and what is the contribution of placental intrinsic defense mechanisms and restriction factors are yet to be demonstrated *in vivo*.

The first evidence of the involvement of dNK cells in controlling viral infection was described for HCMV. Indeed, we have shown that dNK are able to infiltrate HCMV-infected tissue and to co-localize with infected cells. The exposure of dNK cells to HCMV-infected cells was associated with phenotypic changes and the acquisition of a cytotoxic function involving the NKG2D and CD94/NKG2C-E activating receptors (2, 35, 38). The combination of maternal KIR, namely the expression of KIR2DS1, also increases dNK cell cytotoxic response to HCMV-infected HLA-C2^+^ maternal DSC and prevents viral spread and placental pathology (78). However, even if dNK cells are able to clear HCMV infection from the decidual stroma, placental cells are more resistant to NK cell cytotoxicity.

Beside viruses, the fetomaternal interface can be also threatened by other microbial pathogens such as *Listeria monocytogenes* and *Toxoplasma gondii*. The fact that dNK cells, as well as decidual macrophages and dendritic cells, constitutively express the antimicrobial peptide granulysin (37, 107), would suggest their involvement in controlling these infections. However, it is not clear whether dNK cells can destroy the pathogen while sparing infected maternal DSC and fetal trophoblasts.

Collectively, these findings underscore the importance of early activation of dNK cells in reducing and/or preventing the spreading of pathogens to the fetal placenta. However, we still have to further our understanding on (i) whether dNK cell response can be generalized to other TORCH infections, (ii) whether an exacerbated dNK cell responsiveness and/or changes in the microenvironment would maintain fetal development, (iii) what is the role of “trained” dNK cells in viral confinement and/or spreading, and (iv) how the maternal immune system-dNK cells as well as others innate and adaptive cells—manages immunity to infections while promoting fetal development.

## Concluding Remarks

Through their wide secretome, dNK cells play a crucial role in the regulation of tissue homeostasis and optimal fetal development. Lessons from *ex vivo* studies demonstrated that these cells are highly plastic and may overcome the negative control of their lytic functions in order to control viral dissemination to the fetal compartment. However, the molecular and functional basis underlying the transition from a poorly cytotoxic status during healthy pregnancy to a fully active one during viral infection are yet to be revealed. It is clear that inhibitory receptors participate actively to the education of cNK cells and to the acquisition of their effector functions.

In addition to being educated and endowed with high functional plasticity, dNK cells can also develop “innate memory-like.” As basic knowledge expands, we should envision how to exploit these later developments innate immune memory toward the prevention pregnancy disorders. An example within this notion is the established correlation between the recurrence of miscarriages, FGR or preeclampsia and (i) the KIR repertoire skewing, (ii) KIR/HLA-C match or mismatch, and the (iii) changes in the interval between pregnancies and partners. Another open question is whether dNK cells can expand in response to infected cells and generate a “memory-like” response. Such a memory may generate a natural vaccine against viruses and contribute to the control of viral transmission to the fetus. Beyond pregnancy, understanding mechanisms that regulate the plasticity of dNK cells will be helpful to customize NK cell responsiveness in line with therapeutic requirements.

## Author Contributions

The author confirms being the sole contributor of this work and has approved it for publication.

### Conflict of Interest Statement

The author declares that the research was conducted in the absence of any commercial or financial relationships that could be construed as a potential conflict of interest.
